# Executive Function Training in Childhood Obesity: Food Choice, Quality of Life, and Brain Connectivity (TOuCH): A Randomized Control Trial Protocol

**DOI:** 10.3389/fped.2021.551869

**Published:** 2021-02-24

**Authors:** Cristina Sanchez-Castañeda, Sandra Luis-Ruiz, Marta Ramon-Krauel, Carles Lerin, Consuelo Sanchez, Núria Miró, Sònia Martínez, Maite Garolera, Maria Angeles Jurado

**Affiliations:** ^1^Department of Clinical Psychology and Psychobiology, Institut de Neurociències, Universitat de Barcelona, Barcelona, Spain; ^2^Institut de Recerca Sant Joan de Déu, Barcelona, Spain; ^3^Endocrinology Department, Hospital Sant Joan de Déu, Barcelona, Spain; ^4^Paediatric Endocrinology Unit, Consorci Sanitari de Terrassa, Terrassa, Spain; ^5^Diabetes Education Unit, Consorci Sanitari de Terrassa, Terrassa, Spain; ^6^Pharmacy and Nutrition Unit, Consorci Sanitari de Terrassa, Terrassa, Spain; ^7^Neuropsychology Unit, Consorci Sanitari de Terrassa, Terrassa, Spain

**Keywords:** childhood obesity, cognitive training, executive function, quality of life, neuroimaging, magnetic resonance, connectivity, decision making

## Abstract

**Background:** Individuals with obesity are known to present cognitive deficits, especially in executive functions. Executive functions play an important role in health and success throughout the whole life and have been related to food decision-making and to the ability to maintain energy balance. It is possible to improve executive functions through targeted training. This would involve brain plasticity changes that could be studied through connectivity MRI. The general hypothesis of this study is that executive functions training in children with obesity can improve food choices and produce cognitive and neuroimaging changes (structural and functional connectivity), as well as improve emotional state and quality of life.

**Methods:** Randomized controlled double-blind trial with 12-month follow-up. Thirty children with obesity will be randomly allocated into “executive training” (Cognifit with adaptive difficulty + Cogmed) or “control task” group (Cognifit without adaptive difficulty). Both groups will attend 30–45 min of individual gamified training (Cogmed and/or Cognifit systems) by iPad, five times per week during 6 weeks. Cogmed and Cognifit software are commercially available from Pearson and Cognifit, respectively. Participants will receive an iPad with both apps installed for a 6-week use. Participants will also receive counseling diet information via presentations sent to the iPad and will wear a Fitbit Flex 2 tracker to monitor daily activity and sleep patterns. Main outcomes will be cognitive, emotional, food decision, and quality-of-life measures, as well as neuroimaging measures. Participants are evaluated at baseline (T0), after treatment (T1), and 12 months since baseline (T2).

**Discussion:** Longitudinal study with active control group and 3 time points: baseline, immediately after treatment, and 1 year after baseline. Threefold treatment: executive function training, psychoeducation, and feedback on activity/sleep tracking. We will evaluate the transfer effects of the intervention, including emotional and functional outcomes, as well as the effects on neural plasticity by connectivity MRI.

**Trial registration:** This project has been registered in ClinicalTrials.gov (trial registration number NCT03615274), August 3, 2018.

## Introduction

Childhood obesity is one of the most serious public health challenges of the 21st century. Its prevalence has increased at an alarming rate. Globally, in 2016 the number of children with overweight under the age of 5 was estimated to be over 41 million (1). Children with overweight or obesity are likely to stay obese into adulthood and more likely to develop noncommunicable diseases like type 2 diabetes and cardiovascular diseases at a younger age. Overweight and obesity, as well as their related diseases, are largely preventable. Prevention of childhood obesity, therefore, needs high priority (1). Furthermore, childhood obesity has long-term complications such as psychosocial and psychological problems (i.e., depression and low self-esteem) (2, 3).

Weight-loss programs in children have shown limited success, especially at long term (4). A better comprehension of vulnerability factors related to weight gain should provide valuable information for designing more effective treatments (5). In obesity, there is an imbalance between ingested and expended calories (6). On one hand, there is increased availability of energy-dense food that is high in fat; on the other hand, there is a decrease in physical activity. Therefore, novel interventions addressing both aspects of the problem are needed. Guidelines state that “treatment should focus on reducing caloric intake, increasing physical activity, modifying behavior (motivation and readiness to make behavioral and dietary changes), and including family members as active treatment participants” (2).

In addition, individuals with obesity have significant cognitive deficits, especially in executive functions (7). Executive functions are neuropsychological processes including decision-making, inhibitory control, and working memory. All of them are highly implicated in the cognitive control of eating impulses and in the ability to maintain energy balance, as well as in the motivation and adherence to a reduced-energy diet (8, 9). Inhibitory control is the most consistently reported to be impaired, although there is also impairment in reward sensitivity, attention/set-shifting, and working memory (10). Executive impairment also seems to be on the basis of some of the behaviors characteristic of obesity (i.e., increased food intake, disinhibited eating, and less physical activity). Therefore, it is a relevant factor to be considered in weight-loss interventions (11).

The mechanism by which obesity is associated with cognitive function is not clear yet. Smith et al. (12) suggested a bidirectional relationship, where obesity impairs executive functions by affecting cerebral tissue/mechanisms (such as brain inflammation and/or glucose abnormalities), and this impairment in executive functions has an influence in turn on obesity by causing an inability to regulate food intake, plan ahead, and inhibit responses and by promoting impulsive behavior (13, 14). If obesity exerts a detrimental effect on the brain, it is important to prevent obesity during brain development, particularly during the development of executive functions (10) that begins in early childhood and continues in adolescence, and has been attributed to the maturation of the prefrontal cortex (15).

It is possible to improve executive functions through targeted training. This targeted intensive cognitive training induces more effective neural system operations, providing cerebral plasticity (16). Executive functions and working memory training with game elements significantly improved working memory, but also motivation, training performance, and school performance in low-attending children (17, 18).

MRI can be used to assess changes in brain activity induced by training. Several weeks of daily executive function training enhanced working memory–related activity in middle frontal and intraparietal areas (19) and also produced changes in cortical thickness in young adults (20), thus showing that the neural systems underlying executive functions are plastic (21). Brain connectivity changes after training have also been reported in children (17) and adults (22, 23), and could predict body mass index (BMI) (24). However, even if neuroimaging could be a potential biomarker of responsiveness to treatment, changes in connectivity after obesity treatment (including cognitive training) have not been explored yet.

Lastly, children with obesity may experience more psychological disorders (self-concept, anxiety, and depression) (3) and reduced scores in quality of life (QoL) measures than normal-weight children (25). Treating executive deficits while fostering physical activity and promoting healthy nutritional habits may also help to improve QoL and emotional state.

This project has the purpose of evaluating the impact of executive functions training in BMI, food choice, and cognition in children with obesity as well as in their emotional state and QoL. These changes are expected to induce cerebral plasticity that will be measured by MRI connectivity. We hypothesize that children with obesity undergoing the cognitive training program will perform better than active controls in cognitive measures, take better food-related decisions, and consequently, show changes in brain connectivity, emotional state, and QoL measures at the end of the intervention and during the follow-up at 12 months. We also expect them to maintain BMI.

## Materials and Methods

### Study Design

The design is a randomized controlled double-blind trial with 12 months of follow-up. Patients meeting study criteria are randomly allocated according to a sequential number into the “executive functions training” or “control task training” groups after given written informed consent to the coordinator of the project (C.S.C). A sample of the informed consent can be found in Supplementary Material 1. Randomization is stratified by sex, age (9–12 years), and manual dominance. The same person is the one in charge of monitoring training compliance, so the rest of the team is blind to group allocation. All neuropsychological assessments are performed by an independent psychologist (S.L), blinded to group allocation. Patients and families are also blind to the study-group allocation. Because the intervention has no possible side effects, there are no circumstances under which unblinding is permissible. This protocol report follows the SPIRIT and CONSORT reporting guidelines (26, 27).

### Trial Registration Dataset and Protocol Version

The trial has been retrospectively registered in ClinicalTrials.gov (https://clinicaltrials.gov/) on August 3, 2018. The last version of the protocol is the fourth version (March 25, 2020). Table 1 shows the study version history and a summary of the dataset information.

Table 1Trial registration dataset.

**Table:** 

**Data category**	**Information**
Primary registry and trial identifying number	ClinicalTrials.gov NCT03615274
Date of registration in primary registry	August 3, 2018
Secondary identifying numbers	2016-16-10
Source(s) of monetary or material support	Fundació La Marató de TV3
Primary sponsor	Fundació La Marató de TV3
Contact for public and scientific queries	PI: Maria Angeles Jurado Luque, PhD majurado@ub.edu Coordinator: Cristina Sánchez Castañeda, PhD cristina.sanchez@ub.edu Department of Clinical Psychology and Psychobiology Institut de Neurociències University of Barcelona, Barcelona, Spain, 08035
Public title	Executive Function Training in Childhood Obesity: Food Choice, Quality of Life, and Brain Connectivity (TOuCH)
Scientific title	Executive Function Training in Childhood Obesity: Food Choice, Quality of Life, and Brain Connectivity (TOuCH)
Countries of recruitment	Spain
Health condition(s) or problem(s) studied	Childhood obesity
Intervention(s)	Experimental: experimental
	Active comparator: placebo non-adaptive training
Key inclusion and exclusion criteria	Ages eligible for study: 9–12 years Sexes eligible for study: all Accepts healthy volunteers: no
	Inclusion criteria: being obese according to the criteria by Cole et al. (28)
	Exclusion criteria: neurological, psychiatric, or developmental disorder
Study type	Interventional (clinical trial)
	Allocation: randomized intervention model. Parallel assignment masking: double blind (participant, family, investigator, and outcomes assessor)
	Primary purpose: treatment
Date of first enrollment	Estimated for February 2, 2018
Target sample size	30
Recruitment status	Recruiting
Primary outcome(s)	Change from baseline in cognitive, MRI, food choice, and BMI measures at the end of the intervention and follow-up at 12 months
Key secondary outcomes	Change from baseline in emotional state, physical activity, and quality-of-life measures at the end of the intervention and follow-up at 12 months

**Table:** 

**Study records version**		
**Version**	**Submitted Date**	**Changes**
1	July 30, 2018	Original version
2	October 14, 2019	Study status
3	November 21, 2019	Eligibility and study status
4	March 25, 2020	Study status

### Ethics Approval and Consent to Participate

Ethics approval was provided by the University of Barcelona Institutional Review Board (IRB00003099 protocol 122/V/2016), and by the Consorci Sanitari de Terrassa (02-17-503-039) and Sant Joan de Deu Hospital (PIC-02-19) Review Boards. The parents or legal tutors of all participants (in the presence of the children) gave written informed consent in accordance with the Declaration of Helsinki before enrollment. The trial has minimal risk because it has no adverse effects and it is based on commercially available training programs. Cogmed and Cognifit are commercially available from Pearson and Cognifit, respectively. Both programs have previous publications guaranteeing their efficacy (*see next point*). Furthermore, the IRB protocol (IRB00003099) from the University of Barcelona ensures that participation is voluntary, that participants could quit at any time they chose, that signed informed consent is obtained, that confidentiality is maintained, and that data security is rigorous. Participants will receive 20every time they come to the MRI to cover the travel expenses.

Any modifications to the protocol, which may impact on the conduct of the study, potential benefit of the patient or may affect patient safety, including changes of study objectives, study design, patient population, sample sizes, study procedures, or significant administrative aspects, will require a formal amendment to the protocol. Such amendment will be approved by the Ethics Committee/Institutional review board before implementation and notified to the participants and families.

### Cogmed and Cognifit Evidence of Efficacy

According to Simons et al. (29) (pp. 143–148), “Cogmed Working Memory Training has been classified as one of the five commercial cognitive-training programs whose effectiveness had been assessed by several publications. The studies investigating the effects of Cogmed working memory training were undoubtedly the most numerous and best designed (e.g., often including active controls).” In addition, a recent independent meta-analysis by Aksayli et al. (30) evaluated the impact of Cogmed on people's cognitive and academic skills as a function of the type of transfer (near- or far-transfer). They used stricter inclusion criteria than previous meta-analysis (i.e., including a control group and excluding studies with subjective measures of cognitive/academic skills) to ensure a minimum standard of quality. They reviewed 50 independent studies, from a total of 2,761 records examined, and concluded that the training program increased performance on memory tasks immediately post-test (Hedge's *g* = 0.444) and this improvement remained significant after several months, although it slightly decreased (*g* = 0.365 at follow-up). Participants improved the most in those memory tasks whose demands and visual stimuli were very similar to the trained tasks (very-near-transfer; *g* = 0.566 and 0.487 immediately post-test and at follow-up, respectively), while there was a moderate effect on the participants' performance on those memory tasks not included in the training program (lesser-near-transfer; *g* = 0.246, *p* = 0.002 and *g* = 0.176, *p* = 0.005 at post-test and follow-up, respectively).

The Cognifit software has been however less studied. There are no independent studies to date including a sample of children. Therefore, we have assessed its efficacy according to independent studies on adult population. In a study by Siberski et al. (31), the effect of cognitive training vs. computer gaming was evaluated, and results showed that Cognifit significantly improved monitoring, planning, recognition, response time, shifting, spatial perception, visual memory, and visual scanning with medium-sized effects (Cohen's d ranged from 0.58 to 0.72) and naming, visual perception, and working memory with small effect sizes (*d* = 0.44, 0.37, and 0.41, respectively). However, the computer games group also showed an improvement in divided attention (Cohen's *d* = 0.85), inhibition (*d* = 0.67), recognition (*d* = 0.64), and updating (*d* = 0.44). In a study by Verghese et al. (32), assessing the effect of cognitive training on physical activity (concretely on gait speed), participants who underwent Cognifit training had an improvement in gait velocity during normal walking (*p* = 0.05; calculated Cohen's *d* = 0.65) and in walking while talking condition (*p* = 0.002; calculated Cohen's *d* = 1.15). Finally, an independent review by Shah et al. (33) classified Cognifit training program as delivering Level I evidence (at least two well-designed randomized control trials or quasi-randomized studies, one with high quality and the other at least of moderate quality as rated on a risk of bias score (PEDro score) and Cogmed as Level II evidence (at least one well-designed randomized control trials of high quality as rated on the PEDro score).

### Study Sample and Setting

Participants will be children with obesity aged 9–12 years followed by an endocrinologist pediatrician at two hospitals located in the area of Barcelona: Consorci Sanitari de Terrassa and Hospital Sant Joan de Déu. We attempt to include children until we have a sample of 30 children with a complete training and post-training visit. The pediatric assessment will consist of height, weight, waist circumference, blood pressure, puberal state, and personal and familial history. Height and weight will be converted into BMI.

**Figure 1:**
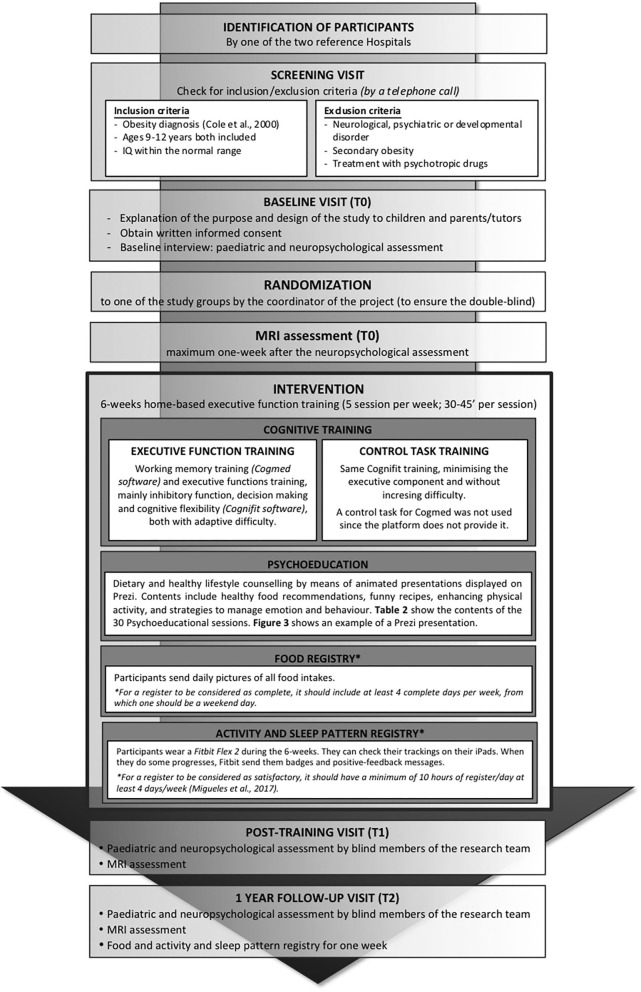
Diagram of the study protocol methods.

**Figure 2:**
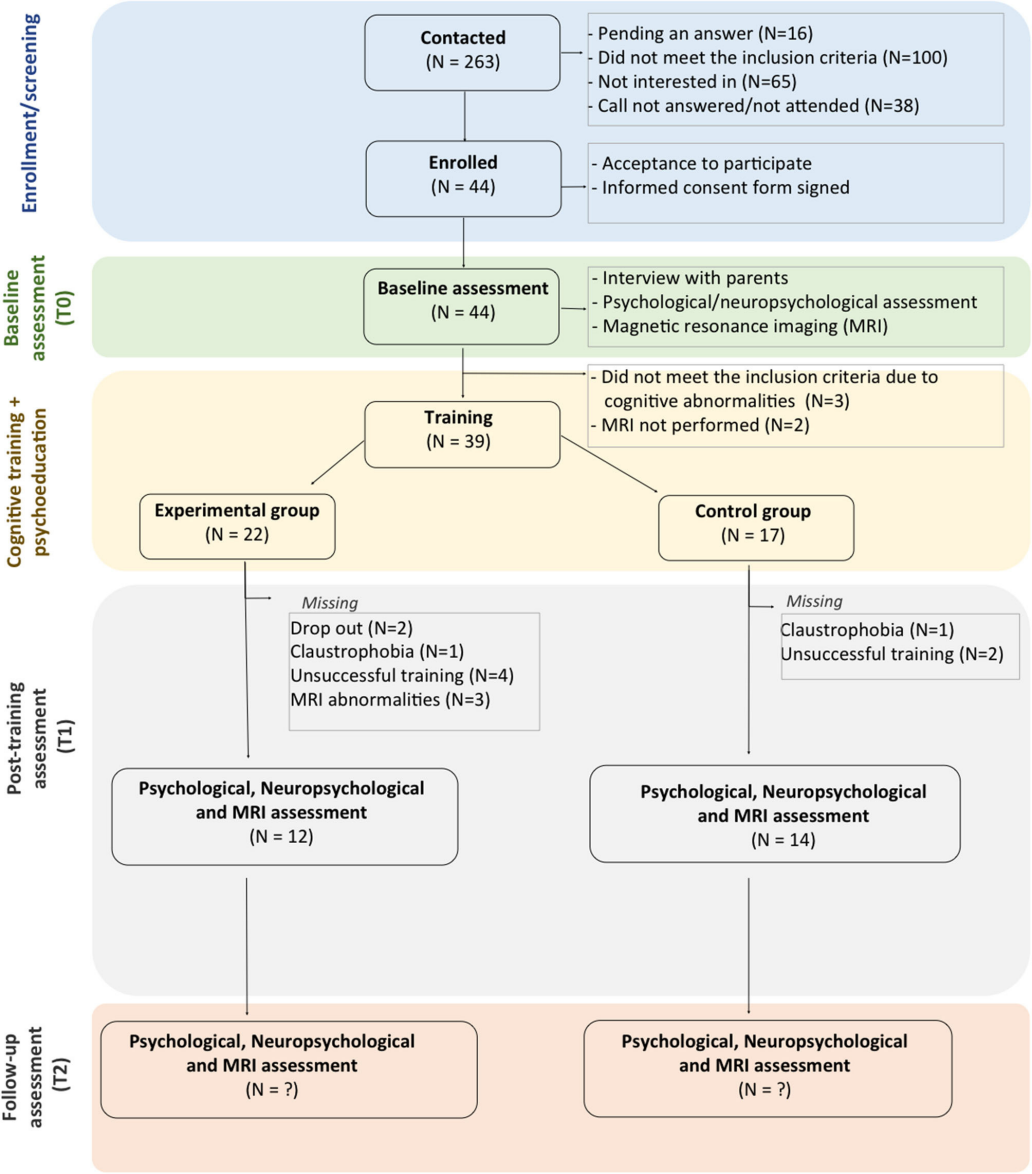
CONSORT flow diagram of study recruitment.

Inclusion and exclusion criteria are detailed in Figure 1. Participants should also have a stable wifi connection at home to be able to perform the training. Figure 2 shows the flow diagram of the study recruitment with the sample recruited to date.

#### Sample Size

We attempt to achieve a total sample size of 30 subjects who have completed the treatment (training rate >70% of the days) and with a complete neuropsychological and MRI post-treatment assessment, 15 children per group. Previous literature on the field has shown significant results with similar sample sizes. Boutelle et al. (34) described a significant decrease in the percentage of eating (*d*_ppc2_ = 0.6, medium effect size) and ingested kcal (*d*_ppc2_ = 0.64, medium size), after training attention in a sample of 14 children (vs. 15 children doing a control task), showing far-transfer effect. Also, Verbeken et al. (35) reported a reduction in BMI (at 2-month follow-up; η_p_2 = 0.16, *d*_ppc2_ = 0.20, small effect) and improvement in short-term visual memory (Corsi block tapping task-forward; η_p_2 = 0.13, *d*_ppc2_ = 0.75, large effect), visual working memory (Corsi block-backwards; η_p_2 = 0.14, *d*_ppc2_ = 0.53, medium effect; BRIEF working memory; η_p_2 = 0.10, *d*_ppc2_ = 0.22), and meta-cognition (BRIEF; η_p_2 = 0.12, *d*_ppc2_ = 0.71) after training 22 children with obesity in visual working memory (vs. 22 children in passive control group). Also, Verbeken et al. (36) showed that an inhibition control and attentional training could significantly reduce inhibition problems (BRIEF; η_p_2 = 0.26, *d*_ppc2_ = 1.91, large effect) in a sample of 22 children with obesity (vs. 15 children in the passive control group).

In addition, multidisciplinary behavioral interventions have shown to produce significant decreases in BMI with similar sample sizes. Delgado-Rico et al. (37) showed a decrease in BMI (Cohen's *d* = 0.5, medium effect size) related with a decrease in impulsivity and inhibitory control in 42 adolescents. Also, Melnyk et al. (38) have shown a significant decrease in weight and BMI (large positive effect size, *d* = 1.5; *p* = 0.05) in a sample of 13 adolescents. Vignolo et al. (39) showed a significant reduction in standardized BMI (calculated Cohen's *d* = 1.75, large effect) and adjusted BMI (calculated Cohen's *d* = 0.66, medium effect) after a hospital-based multidisciplinary program, together with a reduction in waist circumference and improved family habits, emotional and social aspects, and motor skills in a sample of 20 children. Moens and Braet (40) showed a reduction in BMI after 6 months of parent-led cognitive-behavioral training in a sample of 31 children vs. 19 at waiting list [*t*_(30)_ = 2.44, *p* = 0.021]. Finally, Gao et al. (41) assessed the effect of home-based *exergaming* (exercise with videogames) in 18 children compared with an active control group (*n* = 16), showing that cognitive flexibility improved (η_p_2 = 0.19, medium effect size).

Regarding computerized cognitive training, especially those targeting working memory, executive function, and inhibitory control (which are the goal of this study), it may exert a positive effect on cognition assessed with similar sample sizes, concretely in visual, and/or verbal working memory at post-treatment (η_p_2 = 0.21; *d*_ppc2_ = 2.43, large effect size) (17, 42) and at follow-up [η_p_2 = 0.18; *d*_ppc2_ = 3.39, large effect size (42); *d*_ppc2_ = 1.51, large effect (43)], in word fluency (*d*_ppc2_ = 0.57, medium effect), in visuospatial complex memory (*d*_ppc2_ = 0.97–1.08, large effect size) (44), in decision-making (healthy food, hypothetical shopping task) (η_p_2 = 0.118, *d*_ppc2_ = 0.65 and 0.44, medium effect), and in healthy food choice (real food task) (*d*_ppc2_ = 0.65, medium effect) (45).

### Interventions

**Figure 3:**
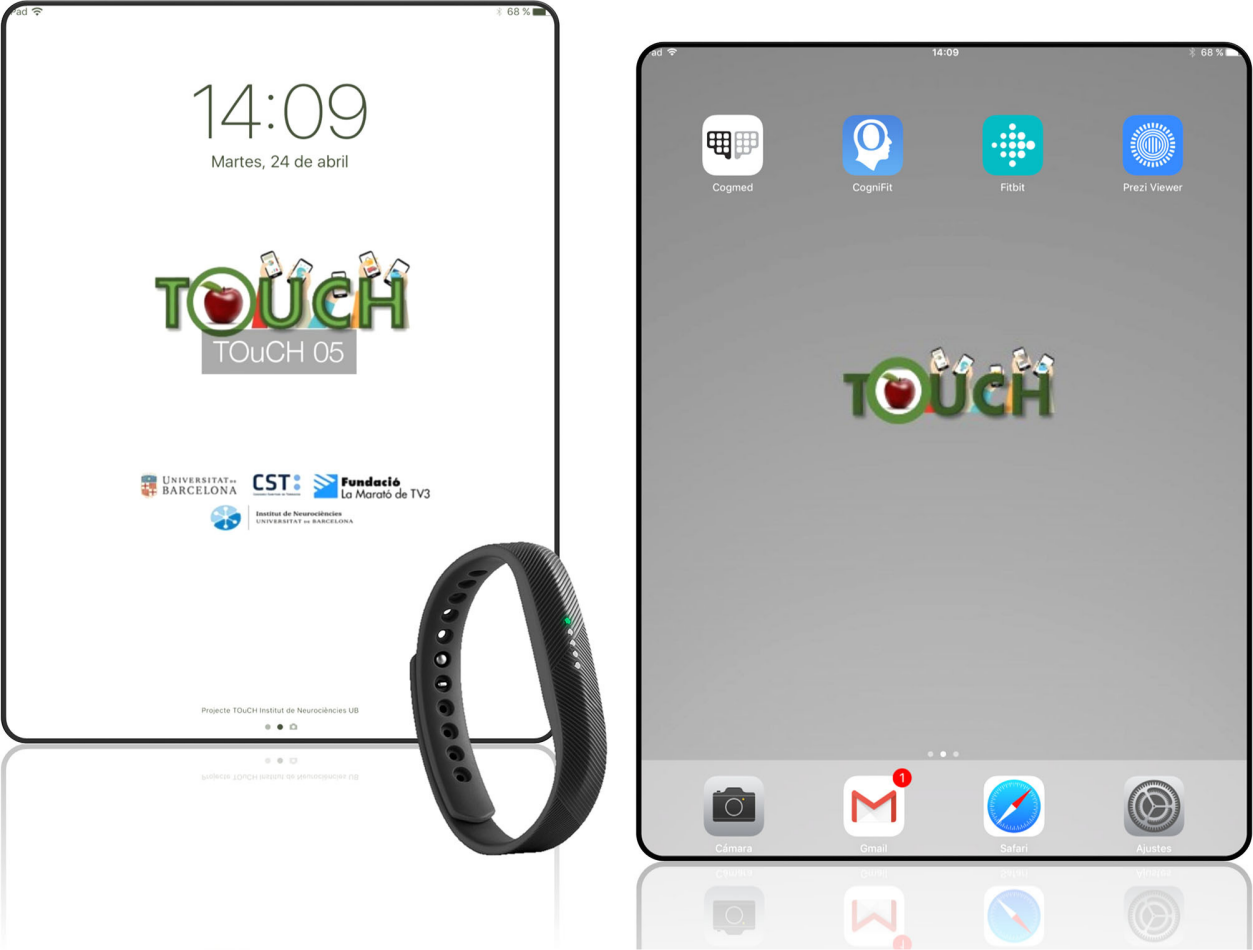
Materials provided to participants to follow the home-based training.

Participants are provided with an iPad (5th generation, wifi) with all the apps needed for the training installed and a mail to contact the study coordinator (who is the only one aware of group allocation) if they have problems. The display of apps and contents in the iPads are remotedly controlled by Zuludesk (now Jamf SCHOOL; https://www.jamfschool.com/). Participants are also provided with a Fitbit Flex 2 (https://www.fitbit.com/flex2) to monitor physical activity and sleep patterns during the weeks of training. Figure 3 shows the materials given to the participants to undergo the training period: an iPad (5th generation) enrolled in the study with all the apps installed and a Fitbit Flex 2.

The coordinator of the study follows the adherence to the training on a daily basis, as well as ensures that participants send and synchronize the required information (pictures of the food intake and Fitbit synchronization). The intervention is threefold (see details in Figure 1).

#### Cognitive Training

All participants undergo a 6-week period of home-based executive function training, five sessions per week, with a duration of 30–45 min per session.

The *executive functions training* consists of working memory training by the Cogmed software (http://www.cogmed.com) and executive functions training (mainly inhibitory function, decision-making, and cognitive flexibility training) by Cognifit software (https://www.cognifit.com), both with adaptive difficulty. These apps are installed in the iPads provided to participants.

The *control task training* will consist of the same training by Cognifit software with non-adaptive difficulty, minimizing the executive component and without increasing difficulty. A control task training for Cogmed was not used because the platform does not provide it anymore.

Participants are asked to perform the training from Monday to Friday. If they cannot perform one or two sessions during the week, they are allowed to make up these sessions during the weekend. In the case of an occasional unfulfillment of the training, we offer the participants to rest 1 week more in training, so they can finish it satisfactorily. In any case, we will extend the training more than 1 week. In the baseline visit with the participants and families, we will also register the number of hours per day/week subjects use to play to other videogames to control for concomitant interventions.

The rest of the procedure will be the same for both groups.

#### Psychoeducation and Food Registry

During the 6 weeks of training, participants are required to send daily pictures of the food intakes to evaluate if the training exerts a change in the eating patterns. We will consider a weekly register as complete, when we have at least 4 days in a week complete, from which one should be a weekend day.

**Figure 4:**
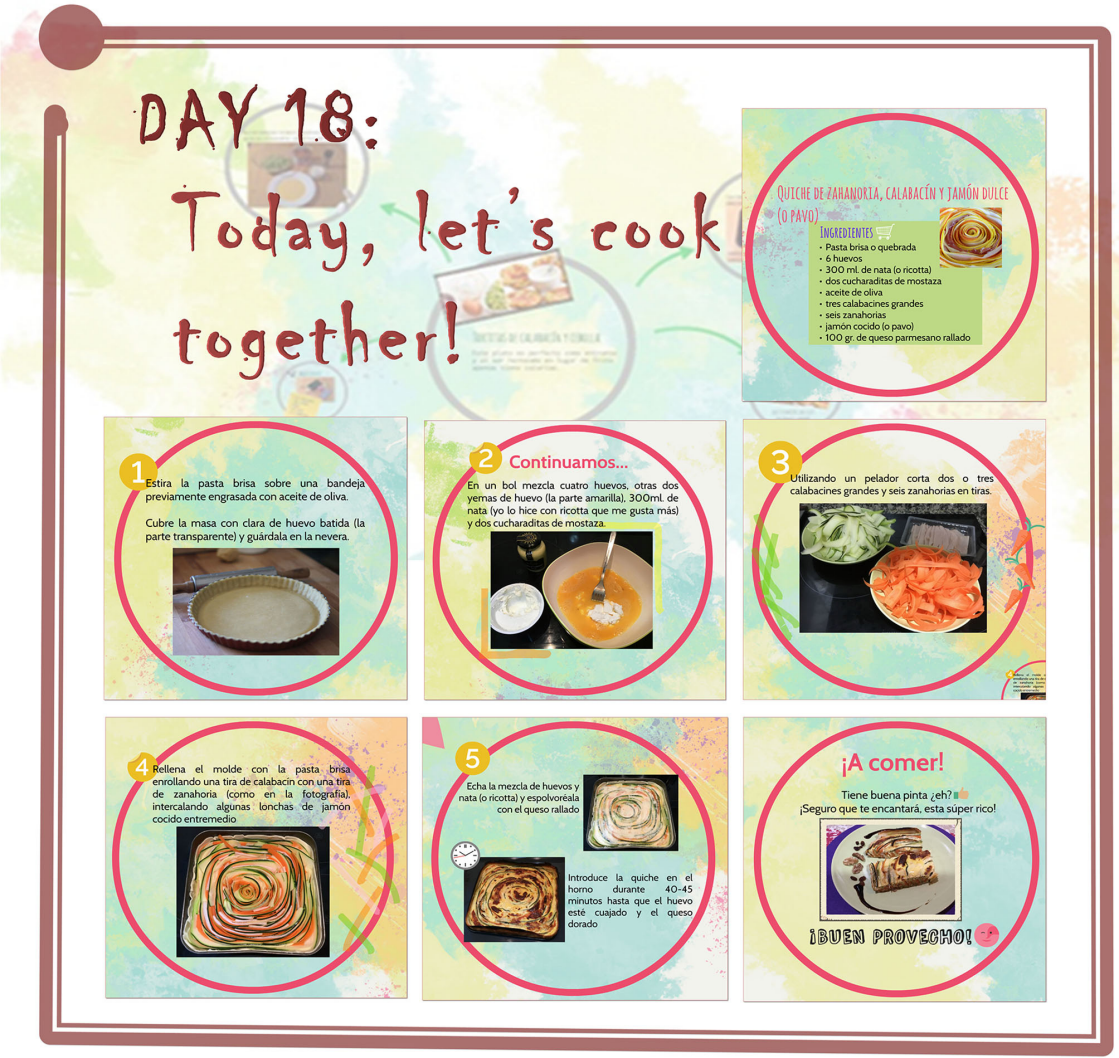
Screenshots of a Prezi daily presentation.

In addition, participants receive dietary and healthy lifestyle counseling by means of animated presentations displayed on Prezi (www.prezi.com). The purpose of those presentations are to encourage participants and their families to learn basic healthy habits by sending them daily presentations with a variety of contents that include healthy food recommendations, funny recipes, enhancing physical activity, and strategies to manage emotion and behavior. Table 2 show the contents of the 30 psychoeducational sessions. Figure 4 shows an example of a Prezi presentation.

**Table 2:** Prezi contents.

	**Prezi contents**
Session 1	Characteristics of a healthy diet (Mediterranean diet)
Session 2	Family activities: help your children to develop healthy habits, “you are a team”
Session 3	Funny colorful recipes
Session 4	Changing your feeding habits: diary goals
Session 5	Introducing new food by playing
Session 6	Buying with responsibility (I)
Session 7	Buying with responsibility (II)
Session 8	Do not snack between meals: just five meals a day!
Session 9	Funny tricks: new recipes
Session 10	Make it easier: weekly menu for planner
Session 11	Going out to a restaurant: some tricks to deal with it
Session 12	Avoid sedentary lifestyle: no more than 2 h with TV and computer
Session 13	Recipe: fish hamburger
Session 14	What do I do if I am nervous?: Strategies to manage your nervousness (I)
Session 15	Eat slow: it can be helpful to your health
Session 16	Physical exercise: a minimum of 60 min per day!
Session 17	Strategies to manage your nervousness (II)
Session 18	Recipe: carrot, zucchini, and York ham (or turkey) quiche
Session 19	Strategies to manage your nervousness (III)
Session 20	Mindful eating: to be conscious on what we eat and how to do it
Session 21	Strategies to manage your nervousness (IV)
Session 22	Recipe: fish meatballs
Session 23	Childhood obesity as a risk factor to develop health disorders
Session 24	Strategies to manage your sadness
Session 25	Recipe: peppers stuffed with potato omelet
Session 26	Funny activities to do on my free time: fighting boredom!
Session 27	Recipe: baked zucchini sticks
Session 28	Positive self-esteem and taking responsibilities!
Session 29	Recipe: zucchini and onion pancakes
Session 30	Last advices and good-bye!

#### Activity and Sleep Pattern Registry

Furthermore, to control for the effect that physical activity and sleep patterns may have on food decisions and BMI, we will register them over the training period by a Fitbit Flex 2. Participants can check their trackings on their iPads. When they do some progresses, Fitbit sends them badges and positive-feedback messages (https://blog.fitbit.com/fitbit-badges/). We consider a register as complete when it has at least 10 h of register/day at least 4 days/week (46).

### Outcomes

We will assess the effect of the intervention on BMI, food choice, global cognition, and neural plasticity. Secondary measures will be emotion/behavior, QoL, and physical activity (see details of the registered protocols in Table 1). There will be three evaluation timepoints: (1) *baseline assessment* (T0); (2) *post-training assessment* (T1), which will be performed after the 6-week training period; and (3) 1-year follow-up assessment (T2), which will be done 12 months after T0, with a leeway of 1 month.

We will collect cognitive, psychological (emotional and quality of life measures), and MRI data in all three time points. Participants' parents will also be interviewed in T0 and T2. Socio-economical data from family, school information, and habits of nutrition and physical exercise from children will be obtained to characterize the sample.

Food, activity, and sleep hours registries will be collected during the 6 weeks of training and for a week at 12-month follow-up.

#### Anthropometric Measures

The pediatric assessment will consist of height, weight, waist circumference, blood pressure, puberal state, and personal and familial history. Height and weight will be converted into BMI.

BMI will be considered a primary measure.

#### Cognitive Assessment

The estimated verbal and visual IQ will be evaluated by the vocabulary and the matrix reasoning subtests of the Wechsler Intelligence Scale for Children—fifth edition (WISC-V Spanish version) (47) to control for potential differences in IQ between groups at baseline.

*Attention and speed* will be measured by the forward digit span and forward picture span (WISC-V), Children's Color Trail Test (CCTT) (48), the Five Digits Test (49), and Conners' Continuous Performance Test 3 (CPT3) (50).

The main subdomains comprising executive functions proposed by Diamond (2013) will be assessed. *Cognitive flexibility* will be evaluated by the Five Digits Test and the Children's Color Trail test (CCTT-2). *Working memory* will be assessed by the backward digit span (WISC-V), the backward picture span of the Weschler Non-verbal Scale (WNV) (51), and by an N-back task. *Inhibitory control* will be assessed by the Five Digits Test and a Go-no-go task. *Planning* will be measured by the Tower of London (52). Moreover, the Behavior Rating Inventory of Executive Function (BRIEF) will be administered (53).

We will consider global cognition as a primary measure. Attention and executive function subdomains will be further analyzed by a *post-hoc* analysis with Bonferroni correction for multiple comparisons.

#### Food Choice Registry

During the training period and for 1 week at 12 months' follow-up, participants will send pictures of the daily intake of food and drinks. These will be collected in tables quantifying the type of food per day and week. We will also administer the Kidmed questionnaire (54) at T0, T1, and T2.

A change in food choice will also be considered a primary measure.

#### QoL and Behavioral Measures

We will assess QoL by the Pediatric Quality of Life Inventory (PedsQL v4.0) (55) that includes parent- and self-reported scales of physical, emotional, social, and school functioning. Children's self-esteem and support perceived from classmates, parents, teachers, and friends is assessed by the Self-Perception and Social Support Profile for Children (56), and anxiety and depression by the Child Behavior Checklist (CBCL), a rating scale assessing behavioral and emotional symptoms in children (57) (www.aseba.org).

Changes in these measures will be considered secondary outcomes, resulting from a significant change in the previous ones (anthropometric and cognitive measures).

#### Connectivity Changes: MRI Assessment

All participants will undergo a 3T brain-MRI acquisition at baseline (T0), after treatment (T1), and after 12 months (T2). The scanning protocol will include structural and functional acquisitions. The MRI sequences will be the following: (1) high-resolution three-dimensional T1-weighted images acquired in the sagittal plane (MPRAGE sequence: TR = 2,400 ms, TE = 3.32 ms, TI = 1,000 ms, 256 slices, FOV = 224 mm; flip angle = 8°; 0.8 mm isotropic voxel); (2) 3D T2-weighted images in sagittal orientation (TR = 3,200 ms, TE = 298 ms, 256 slices, FOV = 224 mm; 0.9 mm isotropic voxel); (3) diffusion tensor imaging sequence (DTI) (single-shot diffusion-weighted EPI, *b* = 1,000 mm^2^/s, 30 directions, TR = 7,700 ms, TE = 89 ms, FOV = 244 mm; 2 mm isotropic voxel). Each direction will be acquired twice (AP and PA) and images will be averaged, improving signal-to-noise ratio; (4) resting state fMRI sequence (gradient-echo echo-planar imaging–EPI sequence) (240 volumes; 40 axial slices; TR = 2,500 ms; TE = 28 ms; FOV = 240 mm; flip angle = 80°; 3 mm isotropic voxel); (5) clinical sequences: a gradient T2 sequence (TR = 518 ms; TE = 20 ms; FOV = 240 mm; flip angle = 20°, voxel = 0.8 mm × 0.8 mm × 3 mm) and an axial FLAIR sequence (TR = 9,000 ms; TE = 96 ms; TI = 2,500 ms, FOV = 240 mm; flip angle = 150°, voxel = 0.9 mm × 0.9 mm × 3mm).

Brain plasticity will be assessed by computing brain connectivity through the analysis of DTI and resting-state fMRI sequences. It will be considered a primary measure. However, its analysis needs to be performed *post-hoc* with multiple comparisons' correction (FEW, FDR). Structural and clinical imaging (T1-, T2-weighted, and clinical sequences) are acquired to ensure that there are no significant baseline differences in brain structure between groups.

### Data Analysis Plan

#### Demographic, Neuropsychological, and QoL Variables

For demographic variables, we will use a descriptive analysis of the variables and a comparative analysis between the training and control groups at baseline. We will use the χ^2^ test (and if this is not applicable, Fisher's exact) for categorical variables and Student's *t*-test (if not applicable, the Mann–Whitney *U*-test) for quantitative variables to check that randomization has successfully distributed the possible confounding variables (age, sex, IQ, manual dominance, socioeconomic, and educational status).

For the evaluation of the effectiveness of treatment on BMI and cognition, we will perform a general linear model for repeated measures analysis including these two variables as primary outcomes. We will perform further *post-hoc* analysis evaluating the effects on cognitive domains and emotional and QoL measures if appropriate. Means comparisons will be performed by Student's *t*-test, if the conditions of application are met and confounding variables have been well-controlled (otherwise, adjusted analyses will be performed by multiple regression). Multiple comparisons will be tested by Bonferroni correction. Effect size will also be calculated according to Hunter and Schmidt (58). All statistical tests will be carried using a two-sided test with the significance level set at 5% with the software Statistical Package for the Social Sciences (SPSS, version 24.0) or R (version 3.6.3).

#### Food Choice

To test our hypothesis that along the training weeks participants will make healthier and more balanced food choices, we will compute the content of the tables and analyze them by linear mixed models for longitudinal data. Multiple comparisons will be tested by Bonferroni correction. We will also assess the difference between groups at 12-month follow-up.

#### Imaging Data

To compute brain connectivity and perform graph-theoretical analysis, we need to preprocess MPRAGE, fMRI, and DTI data sets altogether. Thus, we can perform structural (by probabilistic or deterministic tractography) and functional connectivity analyses, i.e., parcellating the brain into functionally related areas using an atlas [i.e., the AAL atlas by (59)]. The mean time series of the obtained regions' voxels can be used to calculate cross-correlations between them, thus obtaining an *n* × *n* correlation matrix that represents each subject's connectivity profile.

To do that, we will segment the MPRAGE images by the automated FreeSurfer stream (version 5.3; available at https://surfer.nmr.mgh.harvard.edu/) into different cortical regions. With FSL [FMRIB's Software Library, http://www.fmrib.ox.ac.uk/fsl/; tract-based spatial statistics (TBSS) and tractography Diffusion Toolbox (FDT)], we will analyze the DTI dataset. After individual FA map extraction, a probabilistic tracking algorithm is used to estimate white matter pathways connecting different regions of interest. Again, a general linear model, correcting for multiple comparisons across space, will be used to compare differences among groups. Finally, we will preprocess functional images (fMRI) using FSL and AFNI (Analysis of Functional NeuroImages, https://afni.nimh.nih.gov/). An independent component analysis (ICA) can be carried out using tensorial independent component analysis as implemented in MELODIC part of FSL. This enables to obtain a set of independent components and identify the common resting-state functional networks (60).

Brain plasticity will be assessed by *post hoc* analysis with a correction for multiple comparisons (FWE, FDR).

#### Additional Analysis: Non-adherence or Missing Data

We will further analyze if discontinuation affects more one of the experimental groups, and we will also evaluate the baseline properties of the subjects that discontinue or perform the intervention at a non-successful level (reaching a number of essays/training days). Missing behavioral data can be handled by multiple imputation. However, in the case of missing, uncompleted, or artifacted MRI data, we will discard these subjects from the group analysis.

Missed trainings will be addressed by an intention-to-treat (ITT) approach to evaluate if they affect the results. Thus, we will analyze the available data by list-wise deletion without imputing the unknown values.

## Details of the Sample Enrollment to Date

A total of 263 children and their families were contacted to participate in the study from the two areas covered by the two participating hospitals. From those, a total of 44 children and their families (16.73%) consented to participate in the study and underwent the first neuropsychological assessment. A total of 16 families are still pending on taking a decision regarding if participating or not; 100 did not meet the inclusion criteria, 65 families refused to participate in the study, and 38 families did not answer to our calls. From the 44 subjects assessed neuropsychologically, 3 children did not meet inclusion criteria for cognitive impairment, and 2 did not perform the MRI assessment. A total of 39 children were therefore randomly assigned to one of the training groups (88.6% of the ones who consented). To date, there are 22 children with a baseline visit in the experimental group and 17 in the control group. From those, 26 children to date completed the training (task completion more than 70%) and have both neuropsychological and MRI post-treatment assessments (66.6%). Five children more underwent only neuropsychological assessment (a total of 79.5%) due to claustrophobia or because of MRI abnormalities. A total of eight children (20.5%) dropped out or did not reach the minimum training requirements (at least a 70% of completion), six in the experimental group and two in the control group. We explain this higher dropout rate in the experimental group due to the increasing difficulty in the training tasks. This results in a 79.5% retention rate with a training completion of at least 70%.

## Discussion

### Confidentiality and Data Management

Personal information about potential and enrolled participants will only be stored at the trial site and be subject to privacy policy. All data will be treated anonymously (with a trial code) and introduced in a database stored in an institutional server that requires accreditation and password to be accessed. Personal information will not be shared and will be deleted after the trial.

A second blind researcher will ensure accuracy of data in the database and will perform a preliminary analysis of data to ensure it distributes on a normal range. All missing data or outliers will be double checked with the original source of information. Data analysis will only be performed with anonymized data by accredited researchers. After trial publication, trial data will be shared in anonymized form on justified request.

### Discontinuation

A patient may withdraw from the study at any time, at his or her parents' request, for any reason without penalty or loss of benefits. In any case, all patients will be followed up at each assessment time point after discontinuation according to the intention-to-treat principle. In the event of withdrawal of consent, data will be deleted if requested.

### Patient and Public Involvement

A pool of patients and families were involved in the design of psychoeducation materials by participating in an assessment of information needs assessment previous to the development of the study. The results of the needs assessment were used to build the psychoeducation sessions contents. Furthermore, during the training period, children are asked on a daily basis about their satisfaction with the training and the level of compliance and burden of it. Motivation pre-intervention and satisfaction post-intervention are also assessed.

### Dissemination Policy

We will disseminate the results from this study via conference presentations and journal publications. The results of the project will be also presented in a Symposium organized by La Marató de TV3 that the Foundation organizes every year (https://www.ccma.cat/tv3/marato/en/recerca/) addressed to the general audience. Article journal will focus on (1) the post-training effects on the cognitive, emotional, and QoL measures; (2) the post-training effects on MRI connectivity; (3) the post-training effect on food choice; and (4) the 1-year effect on the previous measures. We would expect to find a post-training effect that hopefully will maintain at 1-year follow-up. In case we find that the treatment is effective, we will analyze which elements are the most effective to design further interventional studies and to transfer the effect to the clinics.

## Ethics Statement

The studies involving human participants were reviewed and approved by University of Barcelona Institutional Review Board (IRB00003099 protocol 122/V/2016), Consorci Sanitari de Terrassa Review Board (02-17-503-039), and Sant Joan de Deu Hospital Review Board (PIC-02-19). Written informed consent to participate in this study was provided by the participants' legal guardian/next of kin.

## Author Contributions

CS-C, SL-R, MAJ, and MG contributed to the conception and acquisition of data and will analyze and interpret data. MR-K, CL, CS, NM, and SM contributed to the patients' selection. CS-C contributed to drafting the work and the rest of co-authors to revising it critically. All authors approved the final version of the paper and agreed to be accountable for all aspects of the work and ensure the accuracy and integrity of it.

## Conflict of Interest

The authors declare that the research was conducted in the absence of any commercial or financial relationships that could be construed as a potential conflict of interest.

## Abbreviations

AAL: automated anatomical labeling
AFNI: analysis of functional neuroimages
BMI: body mass index
BRIEF: Behavior Rating Inventory of Executive Function
CBCL: Child Behavior Checklist
CCTT: Children's Color Trail Test
CPT3: Continuous Performance Test, 3rd version
DTI: diffusion tensor imaging
EPI: echo-planar imaging
FA: fractional anisotropy
FDT: FMRIB's diffusion toolbox
FLAIR: fluid attenuation inversion recovery
fMRI: functional magnetic resonance imaging
FSL: FMRIB's software library
ICA: independent component analysis
IQ: intelligence quotient
IRB: institutional review board
MD: mean diffusivity
MELODIC: Multivariate Exploratory Linear Optimized Decomposition into Independent Components
MPRAGE: magnetization-prepared rapid acquisition with gradient echo
PedsQL: Pediatric quality of life
QoL: quality of life
SPSS: Statistical Package for the Social Sciences
T0: baseline assessment
T1: post-training assessment
T2: 12-month follow-up assessment
TBSS: tract-based spatial statistics
WISC-V, Weschler Intelligence Scale for Children: fifth edition
WNV: Weschler Non-verbal Scale.
