# Supplementary material for: Executive Function Training in Childhood Obesity: Food Choice, Quality of Life, and Brain Connectivity (TOuCH): A Randomized Control Trial Protocol
Source: Front Pediatr. 2021 Feb 24;9:551869. doi: 10.3389/fped.2021.551869 (PMC7943482; doi:10.3389/fped.2021.551869)
Supplement: Supplementary file 1 [file Table_1.DOCX]

**INFORMATION SHEET FOR PARENTS**

*(translation from the original in Spanish)*

**Study *“Executive function training in childhood obesity: food choice, quality of life and brain connectivity (TOuCH)”*. Fundació La Marató de TV3**

Dear Sir/Madam:

The Institute of Neuroscience of the University of Barcelona, together with the Consorci Sanitari de Terrassa and the Sant Joan de Déu Hospital of Barcelona is carrying out a study to prevent childhood obesity. This project aims to evaluate the benefits of intensive cognitive training on cognitive functions and food decision-making in children with obesity, as well as its effect on emotional state and quality of life. Additionally, we will assess the cerebral plasticity underlying this cognitive training.

The study consists on a medical visit and a neuropsychological evaluation (including also behavioural questionnaires and quality of life measures) in the Hospital of reference (Consorci Sanitari de Terrassa or Sant Joan de Déu Hospital) and a magnetic resonance imaging (MRI) assessment with a scanner of 3 Tesla in the Clinic Hospital of Barcelona.

Data will be acquired three times: at baseline, after 6-weeks of treatment and after 12-months. Neuropsychological assessment will last about 2 hours at baseline and 1 hour the other visits. MRI protocol will last 30 minutes.

Additionally, participants should undergo a 6-weeks training through videogames installed in an iPad device for 30-45 minutes 5 days/week. Participants should also send daily pictures of food intake and wear a Fitbit Flex 2 to monitor physical activity and hours of sleep. The adherence to the training will be monitored through the iPad on a daily basis.

Personal data will be treated confidentially according to the LOPD 15/1999. In the event of withdrawal of consent, data will be deleted if requested.

Participation in the study is voluntary. Therefore, participants are free to withdraw at any time, without giving any reason, without my medical care or legal rights being affected. In any case, all patients will be followed-up at each assessment time point after discontinuation according to the intention-to-treat principle.

**PARENTS INFORMED CONSENT**

**Study *“Executive function training in childhood obesity: food choice, quality of life and brain connectivity (TOuCH)”*. Fundació La Marató de TV3**

I ____________________________________ (Name and surnames)

| I confirm that I have read and understood the information sheet and have had the opportunity to ask questions | |
| --- | --- |
| I understand that my participation is voluntary and that my son/daughter is free to withdraw at any time, without giving any reason, without my medical care or legal rights being affected | |
| I give consent to the anonymous treatment of my data and the data derived from the MRI exam with a research purpose by University of Barcelona | |
| I agree to take part in the study |  |

Signature Date

Signature Date

**INFORMATION SHEET ABOUT THE MAGNETIC RESONANCE IMAGING ACQUISITION**

We will perform an anatomical high-resolution study to characterize cerebral structures and connections. Afterwards, participants will be asked to rest with eyes open fixing a cross in a screen to measure the cerebral response at rest.

Magnetic resonance imaging (MRI) uses a magnet and radiofrequency waves to acquire images of the brain. This is an ordinary clinical assessment and its efficacy has been proved in different medical areas. There are no side effects related to the exposure to this magnetism or radiofrequency waves. The only risk is that the magnet can attract metals inside the body. Therefore, we will assess for any metallic implant in the body of participants previously to undergo the assessment. In case there are doubts of any potential metal that may involve a risk for the health, participants will be excluded from this part of the study. While performing the assessment, the room will be closed for security reasons.

MRI assessment involves being in a room with a magnet. Participants will be lying in a tight bed and will be introduced into the scanner that is a tunnel of 2,5 meters length approximately for about 30 minutes. Participants will also hear a noise during the exploration that comes from the scanner.
